# Exploration of oxidative stress biomarker and hormonal parameters in female with different causes of infertility

**DOI:** 10.12669/pjms.42.6.14880

**Published:** 2026-06

**Authors:** Sumaira Riffat, Zainub Hanif, Mussarat Ashraf, Mehir un Nisa Iqbal, Rehana Rehman

**Affiliations:** 1 Sumaira Riffat, MBBS, MPhil, Department of Physiology, Sindh Medical College, Jinnah Sindh Medical University, Karachi, Pakistan; 2 Zainub Hanif, MPhil, Department of Biological & Biomedical Sciences, Aga Khan University, Karachi, Pakistan; 3 Mussarat Ashraf, MPhil, Department of Biological & Biomedical Sciences, Aga Khan University, Karachi, Pakistan; 4 Mehir un Nisa Iqbal, MPhil, PhD, Department of Physiology, University of Karachi, Pakistan; 5 Rehana Rehman, MBBS, MPhil, PhD, Department of Biological & Biomedical Sciences, Aga Khan University, Karachi, Pakistan

**Keywords:** Endometriosis, MDA, Oxidative stress, PCOS, SOD2, Unexplained infertility

## Abstract

**Objective:**

Infertility a global health issue linked with untoward psychological and social impacts is due to a number of contributing factors; one of which is oxidative stress. This study aimed to determine the potential association of oxidative stress biomarker malondialdehyde (MDA) and the mitochondrial antioxidant enzyme superoxide dismutase-2 (SOD2) with different etiologies of infertility such as polycystic ovary syndrome (PCOS), endometriosis, unexplained infertility and male factor.

**Methodology:**

This cross-sectional study was conducted at Aga Khan University from December, 2023 to November, 2024 on one hundred infertile women aged 18 till 45 years recruited from Australian Concept Infertility Medical Centre in Karachi. Baseline demographics and body mass index (BMI) were recorded and serum levels of MDA, SOD2, Anti-Mullerian hormone (AMH,) and female reproductive hormones were measured using commercially available ELISA kits. Statistical analyses employed binary logistic regression analysis for association of OS with causes of infertility using univariate and multivariate models, Spearman rank correlations and Kruskal-Wallis tests for correlations and comparison of parameters among different causes of infertility.

**Results:**

Females with PCOS were found to have higher BMI in comparison to male factor and unexplained infertility groups (p<0.05). Serum MDA levels were higher than serum SOD2 levels in PCOS group compared to male factor indicating the elevated lipid peroxidation and redox imbalance.

**Conclusion:**

Among the various infertility aetiologies, women with PCOS demonstrated the most marked oxidative imbalance, characterized by elevated MDA levels and a significant inverse correlation with the mitochondrial antioxidant enzyme SOD2.

## INTRODUCTION

Infertility is defined as “the failure to achieve a pregnancy after 12 months or more of regular unprotected sexual intercourse” according to World Health Organization 2023. The prevalence of infertility in couples ranges from 12.6 to 17.5% worldwide^1^ and approximately 22% in Pakistan, ramified as primary 4% and secondary infertility 18%.^2^

Oxidative stress (OS) shares the pivotal role among the myriad factors that contributes to infertility. OS represents the disequilibrium in reactive oxidation by-products and antioxidant bio molecules. The highly reactive hydroxyl ion can alter DNA bases resulting in strand breaks and DNA damage. An entire spectrum of physiological mechanisms is coordinated by reactive oxygen species (ROS) whereas increase in oxidative species results in the aberration of oocyte mitochondrial function, DNA fragmentation and impaired fertilization and various fertility issues.^3^

Malondialdehyde (MDA) is a reactive bio-molecule yielded by lipid peroxidation of polyunsaturated fatty acid that can measure the degree of OS and free radical-mediated damage. Its elevated level indicates increased lipid peroxidation reflecting incapability of antioxidants in scavenging free oxygen radicals, which can be unfavourable to reproductive health.^4^

Superoxide dismutase 2 (SOD2) is a mitochondrial metalloenzymes that dismutases the reactive superoxide ion converting it to hydrogen peroxide & oxygen.^5^ SOD2 is essential in maintaining cellular redox homeostasis and preventing cellular damage by OS.^6^ Mutation in SOD2 gene is linked to infertility in females.^7^

Prolactin (PRL) plays the pleiotropic role in lactation, growth and reproduction as well it is one of the causes of infertility. The elevated PRL has been implicated in hypogonadotropic hypogonadism and anovulatory infertility by decreasing the gonadotrophin-releasing hormone secretion due to suppression in Kisspeptin.^8^

Anti-Mullerian hormone (AMH) is a potential biomarker for the evaluation of ovarian reserve as it reflects the follicular pool. However, it is implicated in female infertility particularly PCOS as it prevents the assortment of the leading follicle by decreasing the sensitivity of the antral follicles to the FSH.^9^ Luteinizing hormone (LH) and follicle-stimulating hormone (FSH) are key gonadotropins that regulate ovarian function. The LH/FSH ratio greater than 2:1 is used as a diagnostic marker in PCOS showing pituitary ovarian axis dysregulation however, elevated LH/FSH ratios has been observed in approximately 60% of women with PCOS.^10^

On the basis of research done by our group on OS, sirtuin1(SIRT1) and antioxidants in female infertility^11^ this study aims to determine the potential association of OS biomarker MDA and antioxidant mitochondrial enzyme SOD2 in females with infertility due to PCOS, endometriosis, male factor and unexplained causes.

## METHODOLOGY

This cross-sectional study was carried out after approval from ‘The Aga Khan University Karachi form December 2023 to November 2024.

### Ethical Approval:

It was obtained from the Institutional Review Committee’ (ERC 2023-9121-27453; dated December 24, 2023).

The sample size was calculated using OpenEpi, with infertility prevalence as 18–22% in Pakistan^2^, at 5% margin of error and 95% confidence interval estimated at least n=118 samples for this study but due to non-response of participants & data gaps, approximately one hundred infertile women were included in this analysis (n=100). Infertile females aged 18–45 years were recruited from the Australian Concept Infertility Medical Centre (ACIMC) during the clinic visits employing the convenient sampling method. Informed consent was obtained from all participants in English/Urdu. Women with any metabolic or hormonal abnormalities, gynecological cancers, smokers and exposure to radiations or having unsatisfactory general physical health were excluded. Demographic parameters of all participants were noted and all the groups were examined for general health check-ups, height and weight measurements and body mass index (BMI).

The baseline hormonal profile (FSH, LH) was obtained from desk records of ACIMC. All infertile women were matched based on age and BMI. PCOS diagnosis followed ASRM (American Society for Reproductive Medicine) criteria requiring two of three conditions:

- Oligo/anovulation,
- Clinical/biochemical hyperandrogenism, and
- Ultrasound-confirmed polycystic ovaries with ≥12 follicles of 2–9 mm.^12^

Endometriosis in females were diagnosed on the basis of ultrasound reports and laparoscopic findings from ACIMC data record. Females with unexplained infertility were recruited after complete diagnostic workup for both female and male partner including detailed medical history, hormonal/metabolic profile, semen analysis and ovarian reserve assessment.^13^ Male factor infertility was attributed to women having infertility issue in male partner with comprehensive hormonal and semen evaluation showing sperm abnormality.^14^ Females with male factor infertility were included as a comparative group of women having normal reproductive functions but were facing partner based infertility.

Ten milliliters of venous blood was collected on day two of the menstrual cycle. Serum from all females were isolated at 4,000 rpm for 10 minutes and stored at -80°C. Serum MDA, SOD2, PRL and AMH levels were measured using commercial ELISA kits. Statistical analysis was performed using SPSS 23. Statistical analyses employed ANOVA to compare baseline parameters, binary logistic regression analysis for association of OS biomarkers with different etiologies of infertility using univariate and multivariate models, Spearman rank correlations for biochemical parameters and Kruskal-Wallis tests for comparison of parameters with causes of infertility. P value ≤ 0.05 was considered statistically significant with 95% confidence interval.

## RESULTS

Females were grouped according to different causes of infertility. Distribution of females with PCOS was (n=50), Endometriosis (n=20), male factor (n=15) and unexplained infertility (n=15). The comparison of baseline characteristics of infertile females showing significant mean difference for BMI across infertility causes (p=0.046) particularly in PCOS group is presented in Table-I.

**Table-I:** Comparison of baseline parameters in females with different Cause of Infertility.

Parameters	Cause of infertility				p-value
	Endometriosis (n=20)	Male Factor(n=15)	PCOS(n=50)	Unexplained(n=15)	
	Mean ± SD	Mean ± SD	Mean ± SD	Mean ± SD	
Age (years)	31.62 ±4.52	32.47 ±5.03	30.38 ±5.39	33.40 ±4.53	0.079
Weight (kg)	67.86 ± 10.81	65.58 ±15.05	78.90 ±15.77	65.07 ±10.21	0.097
Height (m)	1.56 ±0.06	1.56 ±0.05	1.56 ±0.04	1.56 ±0.04	0.616
BMI (kg/m^2^)	22.11 ±4.46	20.94 ±5.57	33.66 ± 4.17	20.90 ±4.45	0.046*
Duration of Marriage (years)	7.58 ±4.19	10.39 ±5.43	6.74±4.56	8.23 ±4.29	0.057

* p<0.05 :statistically significant using one way ANOVA

The comparison of biochemical parameters among groups of infertility showing that the MDA and LH hold the significant difference across various groups especially in PCOS is depicted in Table-II. The spearman correlation analysis of studied variables with each other showed a significant positive correlation of age with BMI (r=*0.205*,p=0.001*) and LH (r=*0.149*,p=0.019*) whereas negative association with AMH (r=-0.193, p=0.002*). BMI was significantly correlated with MDA (r=*0.375*, p=0.000*) & LH (r=*0.170*,p=0.008*) but negatively correlated with SOD2 (r=-0.169,p=0.008*). MDA showed a significant negative correlation with SOD2 (r=*-0.267*,p=0.000*) while correlated positively with LH (r=0.246,p=0.000*). SOD2 showed significant negative correlations with BMI (r=-0.169,p=0.008*) and MDA (r=*-0.267*,p=0.000*) . OS marker MDA showed a significant positive correlation with BMI while a strong negative correlation is existed with antioxidant SOD2.

**Table-II:** Comparison of biochemical parameters in females with different Cause of Infertility.

Parameters	Endometriosis (n=20)	Male Factor (n=15)	PCOS (n=50)	Unexplained (n=15)	p-value
	Mean ± SD	Mean ± SD	Mean± SD	Mean ± SD	
Malondialdehyde (ng/mL)	36.69 ± 5.44	31.54 ± 4.21	44.33 ± 5.54	38.33 ± 4.32	0.03*
Super Oxide dismutase2 (pg/mL)	19.47 ± 11.1	23.96 ±13.4	17.27± 12.2	18.61 ± 14.6	0.12
Luteinizing Hormone (mIU/L)	5.96 ± 5.46	5.36 ± 5.49	8.61± 5.72	5.43 ± 4.51	0.05
Follicle Stimulating Hormone (mIU/L)	5.09 ± 3.11	5.11 ± 4.55	5.53 ± 4.54	5.02 ± 6.63	0.29
Prolactin (µg/L)	17.43 ± 7.55	16.13 ± 10.1	19.32 ± 8.47	16.37 ±5.85	0.09
Anti-Mullerian Hormone (ng/mL)	3.1 ± 3.42	3.0 ± 6.54	5.27 ±5.18	2.9 ±3.45	0.06

* p<0.05; statistically significant using Kruskal Wallis Test.

The significant association of BMI, MDA, LH and AMH with different causes of infertility whereas SOD2 showed significant negative association are shown in Table-III. BMI, MDA and LH retained their positive association with infertility in different types after adjusting for the remaining predictor whereas SOD2 showed significant negative association suggesting a potential/plausible link to redox modulation in different types of infertility. FSH, PRL and AMH showed positive association but were not independent predictors of cause of infertility.

**Table-III:** Association of Oxidative Stress Biomarkers with different causes of Infertility(Binary Logistic Regression).

Predictors	Univariate Model Odds Ratio (95% C.I)	Multivariate Model Odds Ratio (95% C.I)
Age (years)	1.03 (0.99-1.08)	1.15 (0.82-1.10)
BMI (kg/m2)	1.55* (1.37-1.76)	2.24* (1.61-3.13)
Duration of Marriage (years)	1.01 (0.95-1.06)	1.03 (0.97-1.02)
Malondialdehyde	1.28* (1.21-1.35)	1.44* (1.26-1.63)
Super Oxide dismutase2	0.55* (0.51-0.61)	0.59* (0.52-0.71)
Luteinizing Hormone	1.45* (1.23-1.70)	1.50* (1.01-2.21)
Follicle Stimulating Hormone	1.28 (0.94-1.43)	1.09 (0.84-1.41)
Prolactin	1.03 (0.09-1.77)	1.04 (0.91-1.20)
Anti-Mullerian Hormone	1.34* (1.13-1.59)	1.80 (0.93-3.46)

Dependent variable : Association with Infertility

* p<0.05

## DISCUSSION

The present study illustrated the elevated levels of OS marker MDA in serum of infertile female with PCOS. Higher serum concentrations of OS biomarker MDA in PCOS females supports the findings of various studies pointing the key role of redox imbalance in the pathogenesis of PCOS resulting in chronic inflammation, insulin resistance, follicular dysfunction and dysregulation of pituitary ovarian axis.^15^ Disturbances in redox potential lead to peroxidation of membrane phospholipids, resulting in cytotoxicity that disrupts cell membrane integrity, enzyme structure and function, and induces DNA damage, ultimately culminating in cell death.^15^-^17^

Serum antioxidant SOD2 levels showed nonsignificant variance across various infertile groups. However, SOD2 displays a significant inverse association with serum OS marker MDA and BMI that underpins the potential interplay between metabolic and oxidative factors in different etiologies of infertility as observed in previous studies.^7^,^18^ This finding also suggests that antioxidant enzymatic defense mechanisms may not be proportionally compensated despite the increased OS as observed in infertile group with increased BMI particularly PCOS. Although with small sample size, it could be seen from these findings that an increase in serum MDA or BMI may potentially impair redox milieu or vice versa that might plausibly lead to reproductive pathology, as seen in other studies.^19^ This association can be validated by comparing the biomarkers in healthy fertile females and further investigating the genetic and epigenetic factors in cases and control cohorts.

Regarding hormonal parameters, LH levels demonstrated positive association with BMI and MDA in PCOS group showing higher LH concentration compared to other infertility groups. This elevated LH level found in PCOS group is aligned with the dysfunction of hypothalamic pituitary ovarian axis resulting in altered gonadotropin secretion as seen in other studies.^10^,^20^ However, disturbances in reproductive hormone levels may also be attributed to oxidative stress within granulosa cells, potentially exacerbated by the emotional stress experienced by infertile women.^21^

This study showed statistically non-significant but marginally higher AMH levels particularly in PCOS group which is in accordance to another study.^22^ Furthermore, AMH did not show any significant correlation with MDA or SOD2 however, an inverse correlation with age is observed that can lead to decreased ovarian reserve with advancing age and could be the cause of anovulation.^9^ Although PRL levels showed non-significant variance across infertility groups but its mean concentration was found to be more in PCOS and endometriosis group. Elevated PRL levels may be secondary findings in PCOS and are related to menstrual irregularities and anovulatory cycles due to disruption of Gonadotropin-releasing hormone pulsatility via Kisspeptin one neuron suppression.^8^ Correlation analysis in our study denotes positive nonsignificant association of PRL with MDA while negative association with SOD2 which is in accordance with the study done on ovine granulose cells suggesting that elevated PRL can promote oxidative stress through ROS pathway and mitophagy pathway.^23^

The absence of significant hormonal variation among different infertility groups point towards potential anatomical, immunological or male-specific factors. However, a notable inverse relation of OS biomarker MDA with antioxidant biomolecule SOD2 in infertility groups emphasized the role of redox balance in reproductive well-being in accordance to other research studies.^24^,^25^

Holistically, the present study highlights the role of OS as the potential association factor for infertility as a part of complex redox homeostasis particularly in PCOS affecting the reproductive outcome.

### Strengths:

It includes simultaneous estimation of oxidative stress biomarker MDA and antioxidant enzyme SOD2 in various etiologies of female infertility in Pakistan along with various female reproductive hormones.

### Limitations:

It includes small sample size due to non-response of participants and data gaps, single center and cross-sectional type. Furthermore, unequal grouping and lack of comparative analysis with healthy fertile females limits the generalizability of the results. Additionally, lack of assessment of other oxidative stress markers with confounders precludes causal inference.

## CONCLUSION

Pronounced redox imbalance with significantly elevated levels of MDA and decreased SOD2 in females with PCOS points towards contributory role of oxidative stress in the pathogenic mechanisms leading to infertility. However, further studies involving larger sample sizes and well-defined control groups of fertile women are necessary to better clarify these relationships and validate the clinical significance of oxidative stress across different infertility conditions.

### Future suggestions:

Data with greater sample size, multicenter involvement and comparison with normal fertile female is recommended to validate these findings and to elucidate the potential link of oxidative stress marker MDA and mitochondrial SOD2 as a part of complex redox balance in different causes of female infertility as planned for PhD thesis.

### Disclaimer:

This study is a part of PhD thesis exploring the role of OS and SOD2 in female infertility enrolled in university of Karachi. Generative AI was used for improvement in the sentence structure and grammatical correction.

### Authors Contribution:

**SR:** Conceptualization and design of the study, data acquisition, initial manuscript drafting, and overall responsibility for the integrity and accuracy of the work.

**ZHS:** Data analysis and interpretation, statistical input, and critical revision of the manuscript.

**MA: S**tudy design, laboratory analysis, data collection and write up of manuscript

**MI:** Supervision of data organization, and assistance in manuscript writing and editing.

**RR:** Supervision of bench work, guidance on scientific content and structure, final review and editing of the manuscript. And responsible for the accuracy of the study.
